# Association between TLR2 and TLR4 Expression and Response to Induction Therapy in Acute Myeloid Leukemia Patients

**Published:** 2018-10-01

**Authors:** Mani Ramzi, Abolfazl Khalafi-Nezhad, Mahdiyar Iravani Saadi, Zahra Jowkar

**Affiliations:** 1 Hematology Research Center, Department of Hematology, Medical Oncology and Stem Cell Transplantation, Shiraz University of Medical Sciences, Shiraz, Iran; 2 Hematology Research Center, Shiraz University of Medical Sciences, Shiraz, Iran; 3 Department of Operative Dentistry, School of Dentistry, Shiraz University of Medical Sciences, Shiraz, Iran

**Keywords:** Toll-like receptors, Induction chemotherapy, Acute myeloid leukemia

## Abstract

**Background:** Toll-like receptors (TLRs) are a family of transmembrane pattern-recognition receptors that play a crucial role in the realization of innate and adaptive immune response. TLRs may play a role in tumor development and growth because of expression or up-regulation of functional TLRs in some tumors and tumor cell lines. The participation of TLRs in the pathogenesis of acute myeloid leukemia (AML) remains unspecified. This study aimed to investigate the effect of TLR2 and TLR4 expression in peripheral blood mononuclear cells of AML patients in response to induction chemotherapy.

**Materials and Methods:** Eighty- five patients with newly diagnosed AML were evaluated. Using quantitative reverse transcriptase PCR, the mRNA expression of genes TLR2 and TLR4 was measured before starting and after induction chemotherapy. The differences in the mean expression levels of TLR2 and TLR4 before and after chemotherapy were compared using a paired t-test. The mean expression levels of TLR2 and TLR4 regarding laboratory data were analyzed by one-way ANOVA and Chi-square test.

**Results: **We found that the mRNA expression of TLR2 after induction chemotherapy was significantly lower as compared to before treatment (p=0.001). Also, we found a lower TLR4 gene expression level after chemotherapy as compared to before chemotherapy, albeit it was not statistically significant (p=0.21). Moreover, we observed significantly higher expression of TLR2 and TLR4 in AML-M3 cases compared to non-M3 AML patients.

**Conclusion:** The decreased expression of TLR4 in leukemic samples after induction chemotherapy might indicate a novel potential prognostic role for this receptor, particularly in AML-M3 cases.

## Introduction

 The most common type of acute leukemia in adults is acute myeloid leukemia (AML) which results from the arrest of maturation, the proliferation of clonal neoplastic myeloid hematopoietic precursor cells and accumulation of immature myeloid progenitors or blast cells. The current AML therapeutics have improved the life expectancy of leukemia patients. However, high-dose chemotherapy targeting rapidly dividing cells and not proteins causing maturation arrest has resulted in poor efficacy and high toxicities of the current treatment options^1^. For instance, the cure rate of AML in adult patients younger than 60 years and older than 60 years is 35-40% and 5-15%, respectively^2^. In addition, approximately 50–60% of patients relapse after the current AML therapeutics probably caused by the presence of minimal residual disease ^3^. In the last decade, novel targeted therapeutic strategies such as small molecules that interfere with vital signal transduction pathways and cell cycle regulation and new monoclonal antibodies have been developed to further improve the prognosis of AML^4^^,^^5^. Hence, some efforts have been made to find noninvasive AML biomarkers for classification, diagnosis, disease progression and prognosis^6^. Also, the potential value of specific biomarkers for the prediction of survival, response to therapy, development of novel therapeutic strategies against AML and promising candidates for targeted drug development have been investigated^7^^,^^8^. Recently, the potential role of Toll-like receptors (TLRs) in hematologic malignancies have been explored in some studies ^9^^,^^10^. TLRs are a family of transmembrane pattern-recognition receptors (TLR1-13) that play a crucial role in the realization of innate and adaptive immune response. TLRs activate a number of signaling pathways which lead to the secretion of cytokines and chemokines, drive inflammatory reactions and activate the adaptive immune response to eliminate infectious pathogens and cancer debris ^11^^, ^^12^. TLRs are mainly expressed in human immune-related cells such as T cells, B cells, NK cells, monocytes, neutrophils, dendritic cells and macrophages^11^. Furthermore, TLRs may play a role in tumor development and growth because of expression or up-regulation of functional TLRs in some tumors and tumor cell lines^13^. They up-regulate cellular defense mechanisms and DNA repair genes resulting in increased functional DNA repair ^14^. On the other hand, a microenvironment provided by uncontrolled TLR signaling is necessary for tumor cell proliferation and immune response evasion^13^. Therefore, TLRs may act as a double-edged sword in cancer cells ^13^^, ^^14^. Thus, the potential role of TLRs in the pathogenesis, development and novel therapeutic approaches of hematological malignancies has been explored recently. Major TLRs that have been actively investigated in inflammation and cancer are TLR2 and TLR4 ^15^. Of note, stimulation of cell-surface TLR2 and TLR4 on normal hematopoietic cells leads to differentiation and proliferation of hematopoietic stem cells and myeloid progenitor cells^16^. Higher expression levels of TLRs were observed in the plasma cells isolated from patients with multiple myeloma (MM) compared to plasma cells from healthy donors^17^. Also, an association between TLR2 and TLR4 polymorphisms and cancer risk (particularly for gastric cancer) was shown in previous studies^15^. Modulation of growth and drug sensitivity of hematopoietic malignancies such as chronic lymphoid leukemia and multiple myeloma by TLR stimulation have been shown previously ^17^^,^^18^. Interestingly, it was shown that a wide range of TLRs is expressed in AML cells which may be associated with the pathogenesis and development of AML^19^. For example, TLR8 stimulation has been shown to promote AML differentiation and treatment with TLR8 agonists is suggested as a promising new therapeutic strategy for AML^9^. Moreover, increased expression of TLR2 was observed in AML cells and a promising candidate for targeted drug development is a TLR2-binding cell-penetrating peptide^7^.

However, the participation of TLRs in pathogenesis and development of AML remain unspecified. Thus, this study was conducted to investigate the effect of TLR2 and TLR4 expression on peripheral blood mononuclear cells in response to induction chemotherapy and their relevance as prognostic factors in adult patients with AML.

## MATERIALS AND METHODS

 This study included 85 patients (37 females and 48 males) newly diagnosed with acute myeloid leukemia (AML) using census method. They were referred to Namazi Hospital (Shiraz, Iran) from September 2015 to September 2016. All procedures were in accordance with the Helsinki protocol of 1975 and approved by the Ethics Committee of Shiraz University of Medical Sciences (Shiraz, Iran) (Ethics committee code #94-01-01-11095). The median age of the patients was 46.6 years (range 20-86). The diagnosis of AML was consistent with the French-American-British (FAB)-classification system^20^. After singing the written informed consent by all the patients, bone marrow (BM) samples and peripheral blood (PB) samples (5 ccs) were collected before induction therapy. Ficoll density gradient separation was used to prepare mononuclear cells from the patients. All AML patients, except those with acute promyelocytic leukemia whose regimen was composed of *all*-*trans*-*retinoic acid* (*ATRA*), received standard induction therapy regimen including anthracycline and arabinoside cytosine (daunorubicin 45 mg/m2 on days 1 to 3 and cytarabine 100-200 mg/m2 on days 1 to 7). Morphology and cytochemistry of bone marrow (BM) samples were evaluated and classified according to the French-American-British (FAB) classification system^20^. Complete blood count, blast percentage, and hemoglobin (Hb) level were also determined. After completion of induction treatment, post-induction chemotherapy samples from peripheral blood and second bone marrow samples were obtained on day 21. 

Clinical Response and Complete remission (CR) were defined as neutrophil and platelet recovery to 1•l0^9^ and 100•10^9^/l, respectively, no evidence of extramedullary leukemia, no Auer Rods and <5% blasts in a BM aspirate (irrespective of cellularity) ^21^. 

**Cytogenetic**

Cytogenetic analysis was performed according to the National Comprehensive Cancer Network (NCCN) guidelines ^22^. A favorable abnormality was defined by the presence of mutated (CCAAT/enhancer binding protein-alpha) CEBPA or (nucleolar phosphoprotein B2) NPM1 without FLT3-ITD, or t (8; 21), t (15; 17) or inv/t (16). Patients were considered to be at intermediate risk if normal cytogenetics, trisomy 8, t (9; 11), t (8; 21), inv (16), t (16; 16) with the cKIT mutation were diagnosed. NCCN guidelines categorize a poor risk abnormality by the presence of t11q23 [other than t (9; 11)], del7/7q aberrations, del5/5q, t (6; 9), inv3, t (3; 3) aberrations or a complex karyotype (three or more numerical and/or structural abnormalities) and normal cytogenetics with FLT3-ITD mutation. 

**Ribonucleic acid isolation and cDNA synthesis**

Five ml of peripheral blood from each patient was collected in ethylenediaminetetraacetic acid (EDTA)-containing tubes at the time of diagnosis prior to chemotherapy and after chemotherapy. The peripheral blood mononuclear cells (PBMCs) were isolated from each individual using Ficoll-hypaque density gradient centrifugation. Total RNA was isolated by Trizol (Invitrogen, Carlsbad, CA) using the RNX-Plus solution (CinnaGen, Tehran, Iran) according to the protocol supplied by the manufacturer. The quantity and quality of extracted RNA were determined by Nanodrop (Thermo Fisher Scientific, USA) (measuring the optical density 260/280) and by running 3 μL on 1% agarose gel, respectively. The quality of RNA was indicated by the presence of 28S ribosomal RNA twice as intense as the 18S rRNA and the lack of a smear on the lower part of the gel (a smear indicates RNA degradation). After obtaining good-quality total RNA, cDNAs were prepared with reverse transcription (Prime Script RT Reagent Kit, Takara, Shiga, Japan) according to the manufacturer’s guidelines using a T100 thermal Cycler (Bio-Rad Laboratories, USA) and mRNA-specific stem-looped primers. 

**SYBR green real-time polymerase chain reaction (PCR)**

The relative mRNA expression of genes TLR2 and TLR4 was evaluated by the SYBR Premix Ex Taq II (Tli RNaseH Plus) Master Mix (Takara, Japan). Internal control for minor fluctuations was Glyceraldehyde 3-phosphate dehydrogenase gene and the expressions of TLR2 and TLR4 mRNAs were normalized. Polymerase chain reaction program and primer sequences are presented in Table 1. A melting curve analysis was performed at the end of each run to confirm the specificity of amplification. In the absence of primer dimer, fluorescence data for the target genes were collected and normalized to the internal standard gene GAPDH. The relative quantitation of each target gene expression was indicated by cycle threshold (Ct) values which were inversely proportional to the original relative expression level of the gene of interest. To obtain the relative expression level of target genes, (2–ΔΔCT) method was used where ΔΔCt = [ΔCt (before chemotherapy) - ΔCt (after chemotherapy)] and ΔCt= [Ct (sample) –Ct (housekeeping gene)].

**Table 1:** The Primers and PCR condition for the TLR4, TLR2 and GAPDH gene

**Gene**	**Primer sequences (5'->3')**	**Thermocycling ** **condition**
TLR4: F	CAAGAACCTGGACCTGAG	95 ͦ C/2 min, 40 cycles of 95°C/30 sec,
TLR4: R	TGGATTTCACACCTGGATAA	58°C/20 sec and 70°C/30 sec
TLR2: F	TTGCTTACTTCCTAGTCC	95 ͦ C/2 min, 40 cycles
TLR2: R	TCACTTGGTCACTAAGAG	of 95°C/30 sec, 58°C/20 sec and 70°C/30 sec
GAPDH: F	GGACTCATGACCACAGTCCA	95 ͦ C/2 min, 40 cycles
GAPDH: R	CCAGTAGAGGCAGGGATGAT	of 95°C/30 sec, 57.5°C/20 sec and 70°C/30 sec

**Statistical analysis**

The results were statistically analyzed using SPSS software, version 15. The expression levels of TLR2 and TLR4 were compared between patients according to response to chemotherapy treatment, type of AML (FAB subtypes) and cytogenetic aberration by independent t-test. The differences in the mean expression levels of TLR2 and TLR4 before and after chemotherapy were compared using the paired t-test. Also, Chi-square test and one-way ANOVA were used for analyzing the mean expression levels of TLR2 and TLR4 regarding laboratory data. P-values less than 0.05 were considered significant. 

## Results

 The relative mRNA expression levels of TLR2 and TLR4 obtained from 85 AML patients (48 males and 37 females) were evaluated by real-time PCR. The clinical and laboratory characteristics of AML patients are presented in Table 2. Thirty-six (42.4%) out of 85 patients were diagnosed as M2, 13 (15.3%) M4, 12(14.1%) M5, 10(11.8%) M3, 12 (14.1%) M0 and 2(2.4%) patients belonged to M6 based on the French-American-British (FAB)-classification.

No significant differences were found between mean TLR4 or TLR2 gene expression before chemotherapy and age (p=0.78), sex (p=0.44), Hb levels (p=0.288), blast percentage (p=0.51), WBC count (p=0.26), platelet count (p=0.38) and LDH (p=0.25).

**Table 2:** Patient characteristics

**Sex**	**No. of patients (%)**
Male	48 (56.5)
Female	37 (43.5)
FAB classification	
M0	12 (14.1)
M2	36 (42.4)
M3	10 (11.8)
M4	13(15.3)
M5	12(14.1)
M6	2(2.4)
M7	0 (0)
Median age (years)	46.6(range 20-86)
Blood count	
WBC (× 106)	38.5 (range 0.9-300)
HB	(g/dl) 8.1 (range 3.8-14.7)
PLT	(× 106) 51 (range 3-380)
Response rate after treatment	
Patients with CR response	45 (52.9)
Patients with	NCR response 40 (47.1)
Smear (% blasts)	
PB	63 (range 18–90)
BM	67 (range 20–98)
Cytogenetic/molecular risk	
Favorable risk	20(23.52)
Intermediate risk	37(43.52)
Poor risk	28(32.94)

CR: complete remission, FAB: French-American-British, HB: hemoglobin, NR:no remission, PLT: platelets

**Change in TLR2 and TLR4 expression in** **AML patients following chemotherapy**

**Figure 1:**
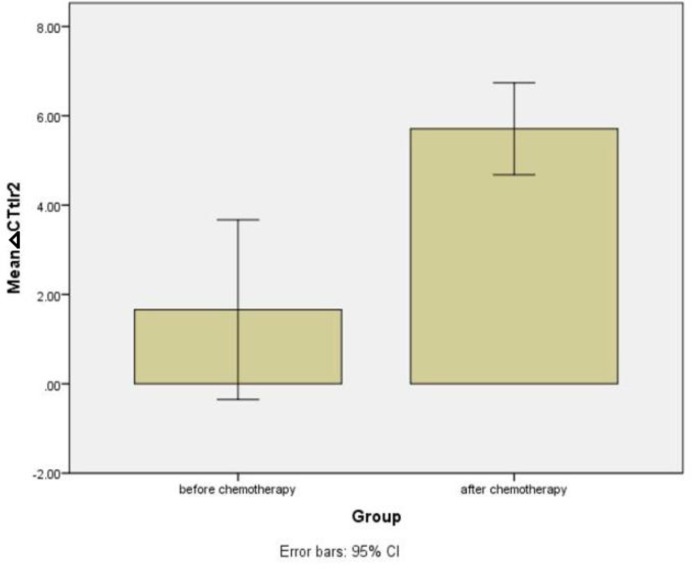
changes in TLR2 expression following chemotherapy

The mean delta Ct for mRNA expression of TLR2 before chemotherapy was 1.65 ±1.01. After chemotherapy, there was a marked decrease in the mRNA expression of TLR2 (ΔCt: 5.7±0.51) compared to its baseline value (P=0.001) (Figure 1).

**Figure2:**
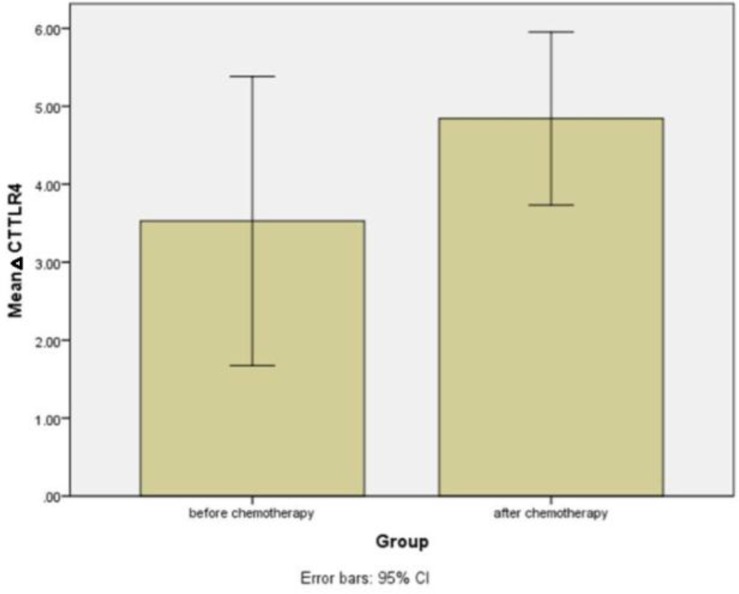
changes in TLR4 expression following chemotherapy

We noted a lower TLR4 gene expression level after chemotherapy (mean delta Ct= 4.84±0.55) compared to before chemotherapy (mean delta Ct= 3.52±0.93), albeit it was not statistically significant (p=0.21) (Figure 2) (Table 3).

**Table 3:** Change in TLR2 and TLR4 expression in AML patients following chemotherapy

	**Before** **chemotherapy** **(x ± SD)**	**After** **chemotherapy** **(x ± SD)**	**P**
**ΔCt TLR2**	1.65 ±1.01	5.7±0.51	0.001
**ΔCt TLR4**	3.52±0.93	4.84±0.55	0.21

X: mean, SD: standard deviation

**TLR2 and TLR4 expression in** **AML patients and response to treatment**

We found that the mean values of the delta Ct for TLR2 in the CR group and the non-CR (NCR) group were 1.1±1.77 and 2.11±0.91, respectively. However, the expression of TLR2 did not show a significant difference between the CR and the NCR groups (p=0.628). 

Expression of TLR4 appears to be lower in the CR (complete response) group (mean delta Ct= 3.77±0.8) compared to the non-CR (NCR) group (mean delta Ct= 3.37±1.65) as indicated by higher mean delta CT value of the NCR group; however, it was not confirmed by statistical analysis (p=0.13) (Table 4). 

**Table 4:** TLR2 and TLR4 expression in AML patients and response to treatment

	**CR** **(x ± SD)**	**NCR** **(x ± SD)**	**P**
**ΔCt TLR2**	1.1±1.77	2.11±0.91	0.628
**ΔCt TLR4**	3.77±0.8	3.37±1.65	0.13

X: mean, SD: standard deviation

**TLR2 and TLR4 expression in AML M3 compared to other types of AML**

**Figure 3:**
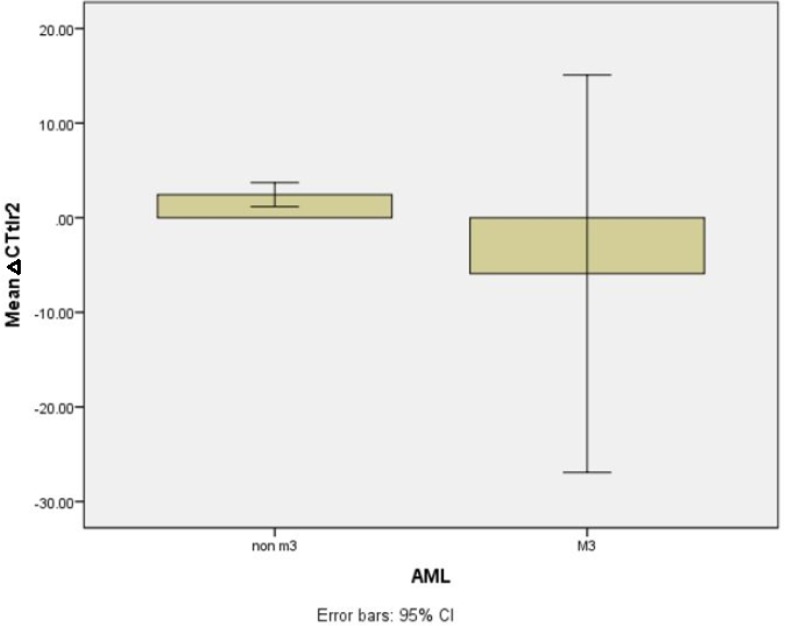
TLR2 expression level in AML M3 and non-M3

We could observe a higher expression of TLR2 in AML-M3 cases compared to non-M3 AML patients (the mean values of the delta Ct in AML-M3 vs. non-M3, -5.9±8.88 vs. 2.44±0.63, P = 0.015) (Figure 3).

**Figure 4:**
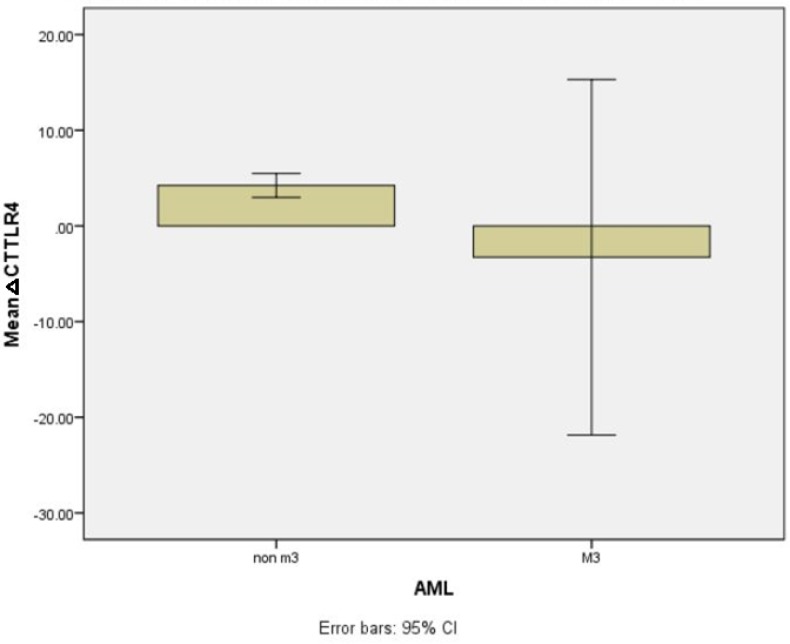
TLR4 expression level in AML M3 and non-M3

Also, a higher expression of TLR4 in AML-M3 cases was observed compared to non-M3 AML patients (the mean values of the delta Ct in AML-M3 vs. non-M3, -3.27±7.85 vs 4.23±0.63, P = 0.0001) (Figure 4) (Table5).

**Table 5:** TLR2 and TLR4 expression in AML M3 compared to other types of AML

	**Other types of** **AML** **(x ± SD)**	**AML M3** **(x ± SD)**	**P**
ΔCt TLR2	2.44±0.63	-5.9±8.88	0.015
ΔCt TLR4	4.23±0.63	-3.27±7.85	0.0001

X: mean; SD: standard deviation

**TLR2 and TLR4 expression according to Cytogenetic abnormalities status groups**

Delta Ct values for TLR2 mRNA expression levels in favorable (14 cases), intermediate (37cases) and poor (26 cases) risk groups were 0.79±1.6, 2.6±0.92 and 0.65±2.8, respectively. The expressions of TLR2 did not show significant differences in risk groups (p=0.67).

Delta Ct values for TLR4 mRNA expression levels in favorable, intermediate and poor risk groups were 4.01±1.68, 3.44±1.93 and 3.84±0.91, respectively. The expression of TLR4 was not different in favorable vs. intermediate vs. poor risk groups (p=0.97). 

## Discussion

 The aim of this study was to analyze the relationship between the expression of TLR2 and TLR4 and response to induction chemotherapy in AML patients to explore their possible roles as prognostic markers for patient risk stratification at first diagnosis. We found that the mRNA expression of TLR2 after induction chemotherapy was significantly lower as compared to before treatment (p=0.001). Also, we observed a lower TLR4 gene expression level after chemotherapy as compared to before chemotherapy, albeit it was not statistically significant (p=0.21). Moreover, we observed significantly higher expression of TLR2 and TLR4 in AML-M3 cases compared to non-M3 AML patients.

TLRs participate in recognition of broad pathogen-associated molecular patterns, activation of the inflammatory and immune responses leading to the secretion of proinflammatory cytokines such as tumor necrosis factor alpha (TNF-a), interleukin-1 (IL-1) and interleukin-8 (IL-8)^23^. Many endogenous and exogenous factors regulating cell proliferation and survival play critical roles in cancer development. There are TLRs among the regulators of cell proliferation and survival which play a crucial role in the realization of innate and adaptive immune response^11^^,^^12^^,^^24^. The expression level of TLRs may depend on the environment, subset, cell type, stimulus and probably age group^25^. Renshaw et al. have previously reported that aging could have negative effects on TLR expression and function, and therefore leads to increased susceptibility to infections and poor adaptive immune responses^26^. However, we did not find any significant effect between aging and the expression of TLRs. Moreover, some previous studies demonstrated that TLR expression increased in leukocyte from patients with sepsis^27^^,^^28^. Therefore, we excluded patients with sepsis diagnosis in the present study. 

TLR stimulation plays a strategic role in the normal hematopoietic differentiation. Specifically, TLR2 or TLR4 stimulation of normal hematopoietic cells are linked to the stimulation of hematopoietic stem cell and myeloid progenitor cell growth and differentiation which consequently leads to increased myeloid cell counts. Moreover, TLR7/8 have been found to promote the CD34+ differentiation into macrophages and dendritic cell precursors ^29^^,^^30^. 

It has been previously reported that TLRs are expressed by myeloid leukemia cell lines^19^. Also, TLR stimulation can exert direct anti-cancer activity as TLR stimulation plays a role in the activation of immune cells which finally leads to killing cancer cells. TLR agonists have been shown to promote anti-cancer effects through T cell activation. Furthermore, TLRs activated by bacterial products such as lipopolysaccharide (LPS) could perform antitumor effects through cytokine induction^31^. The major TLRs being actively investigated in inflammation and cancer are TLR2 and TLR4 which are expressed on the cell surface^9^. TLR2 plays an important role in recognition of microorganisms and activation of the immune response. TLR4, in association with coreceptor CD14, recognizes LPS ^23^. It has been found that TLR4-induced myeloid/monocytic differentiation of the immature progenitor cells acts as a hematopoietic regulatory system supplying mature myeloid cells in response to infections^32^. Importantly, TLR agonists have shown significant promise for the treatment of cancer due to their immunomodulatory effects. For example, the TLR7/8 ligands have been clinically applied in the treatment of skin cancers and actinic keratosis^33^. TLR agonists have been proposed to strengthen effectively the response of the immune system in leukemia ^25^. 

Li et al. evaluated the expression of TLR2 in AML cells. They found that TLR2 is a potential therapeutic target for the prevention and treatment of AML, and they suggested the prototype, Pep2-D(KLAKLAK)2 as a promising drug candidate in the treatment of AML ^28^.

Beck et al. evaluated the effects of TLR agonists on the maturation and function of 3-day dendritic cells from AML patients in complete remission. They concluded that TLR-matured dendritic cells had clinical application in patients with AML for activation of innate and adaptive immune responses ^34^.

There are few reports regarding the potential roles of TLR2 and TLR4 in the pathogenesis of leukemia. The enhanced TLR2 expression has been already observed in human AML cells in a previous study. It has been reported that TLR2 expression has been also increased slightly in CML, ALL and CLL cells. However, enhanced TLR2 expression was not found in other cells from the bone marrow or lymph nodes. Therefore, TLR2 expression levels have been suggested as a potential prognostic biomarker for AML and it can be used as a potential target for the development of novel therapeutic strategies against AML^7^. Also, genetic variation in the TLR10–TLR1–TLR6 region has been associated with non-Hodgkin lymphoma and some inflammatory diseases such as asthma and Aspergillus infection after allogeneic stem cell transplantation^35^^-^^37^. Additionally, the TLR2 gene variant has been linked to decreased risk of chronic lymphocytic leukemia (CLL) and increased risk of follicular lymphoma (FL)^38^. TLR2 also may initiate some immune response to H. pylori and consequently participate in the pathogenesis of gastric MALT lymphoma^39^. Lower expression levels of TLR1, TLR3, TLR4, TLR7, and TLR9 have been found in peripheral blood mononuclear cells (PBMCs) from patients with ALL compared with those from control patients in a previous study ^25^.

We detected mRNA expression levels of TLR2 and TLR4 in 85 patients with AML before the beginning of treatment and compared them with mRNA expression of TLR2 and TLR4 after chemotherapy. Lower mRNA expression levels of TLR2 and TLR4 were observed after induction chemotherapy as compared to before treatment, albeit TLR4 expression level was not statistically significant (p=0.21). Complete remission was achieved in 52.9% of the patients (n=45). There have been only a few reports on the analysis of TLR potential role in the development of AML. Sánchez-Cuaxospa et al. have explored the expression levels of TLRs in AML patients and found lower expression levels of TLR1, TLR3, TLR4, TLR7 and TLR9 in the PBMCs of ALL patients compared with those from control patients. Also, in a study by Webb et al. the TLR4 expressions in lymphocytic leukemias and myeloid leukemias were decreased compared to normal controls^40^. They have attributed the decreased expression of TLR4 to an impaired host response at various stages towards the malignant clonal populations and supposed that the lack of sufficient host TLR4 could possibly lead to depressed resistance to the challenge of leukemic transformation^40^. The reported discrepancy between the results of the present study and that of Webb et al. may be related to the different populations which were investigated. Moreover, we observed a higher expression of TLR2 and TLR4 in AML-M3 cases compared to non-M3 AML patients. It has been proposed that the high expression of TLRs in patients without response after therapy be correlated with the stimulation of immune mechanisms enabling leukemic cells to survive and lead to the promotion of carcinogenesis^24^. The results of our study are in line with some previous studies concerning the role of TLR2 and TLR4 expression levels in AML. Rybka et al. reported that Lower TLR2 and TLR4 mRNA expression levels were found in AML patients with complete remission after the first cycle of chemotherapy compared with patients without response to treatment. Unlike the present study in which AML-M3 cases had a higher expression of TLR2 and TLR4 compared with non-M3 AML patients, in the study by Rybka et al. the higher expression of TLR2 and TLR4 was found more in patients with myelomonocytic and monoblastic acute leukemia than in other types of AML^24^. 

It is hardly possible to compare our results with those of other authors because the exact role of TLRs in the development of AML remains unclear. It seems that TLRs may play a “double-edged sword” role in cancer cells. On the one hand, they up-regulate cellular defense mechanisms and DNA repair genes resulting in increased functional DNA repair^14^ and on the other hand a microenvironment provided by uncontrolled TLR signaling is necessary for tumor cell proliferation and immune response evasion^13^. Positive TLR2 expression in the tumor microenvironment is supposed as an indicator of activated immune surveillance against the altered epithelial cells, whereas TLR2 expression by malignant keratinocytes is suggestive of resistance to apoptosis as a prosurvival mechanism^41^. Induced resistance to TNF-related apoptosis-inducing ligand (TRAIL)-induced apoptosis in tumor cells and enhanced secretion of immunosuppressive cytokines have been attributed to TLR4 ligation on tumor cells ^42^^,^^43^. In contrast, promoted tumor cell adhesion and invasion in a murine model have been reported to be associated with lipopolysaccharide (LPS) ligation to TLR4^44^ and increased tumor progression and metastasis have been correlated with the silencing of TLR4 in a murine model of breast cancer^45^^.^

Although our results show lower expression of TLR4 after induction therapy, further studies should be conducted to address the expression of TLRs in different mononuclear cell types. Variation between cell populations should be assessed to determine probable different levels of expression in different types of mononuclear cells and/or subtypes of AML. The present study had some limitations. We did not analyze prognostic parameters like overall survival. Additional clinical investigations of longer follow-up time and the larger size may be needed in future studies addressing the prognostic value of TLR expression levels in AML. Also, the probable role of TLRs in consolidation therapy and the outcome of allogeneic hematopoietic stem cell transplantation should be explored in future studies. The total population of leukocytes has been evaluated in the current study and further studies focusing on evaluating TLR2 and TLR4 gene expression in more narrowly defined cellular subsets should be conducted.

## CONCLUSION

 Based on the results of this study, it can be concluded that the decreased expression of TLR4 in leukemic samples after induction chemotherapy might indicate a novel functional role for this receptor, particularly in AML-M3 cases. However, this observation should be validated by a larger study. 
